# AhRR and PPP1R3C: Potential Prognostic Biomarkers for Serous Ovarian Cancer

**DOI:** 10.3390/ijms241411455

**Published:** 2023-07-14

**Authors:** Alessandra Ardizzoia, Andrea Jemma, Serena Redaelli, Marco Silva, Angela Bentivegna, Marialuisa Lavitrano, Donatella Conconi

**Affiliations:** School of Medicine and Surgery, University of Milano-Bicocca, 20900 Monza, Italy; a.ardizzoia@campus.unimib.it (A.A.); a.jemma@campus.unimib.it (A.J.); serena.redaelli@unimib.it (S.R.); marco.silva@unimib.it (M.S.); angela.bentivegna@unimib.it (A.B.); marialuisa.lavitrano@unimib.it (M.L.)

**Keywords:** ovarian cancer, copy number alterations, cancer stem cells (CSCs), AhRR, PPP1R3C, array-CGH, prognostic biomarkers

## Abstract

The lack of effective screening and successful treatment contributes to high ovarian cancer mortality, making it the second most common cause of gynecologic cancer death. Development of chemoresistance in up to 75% of patients is the cause of a poor treatment response and reduced survival. Therefore, identifying potential and effective biomarkers for its diagnosis and prognosis is a strong critical need. Copy number alterations are frequent in cancer, and relevant for molecular tumor stratification and patients’ prognoses. In this study, array-CGH analysis was performed in three cell lines and derived cancer stem cells (CSCs) to identify genes potentially predictive for ovarian cancer patients’ prognoses. Bioinformatic analyses of genes involved in copy number gains revealed that AhRR and PPP1R3C expression negatively correlated with ovarian cancer patients’ overall and progression-free survival. These results, together with a significant association between AhRR and PPP1R3C expression and ovarian cancer stemness markers, suggested their potential role in CSCs. Furthermore, AhRR and PPP1R3C’s increased expression was maintained in some CSC subpopulations, reinforcing their potential role in ovarian cancer. In conclusion, we reported for the first time, to the best of our knowledge, a prognostic role of AhRR and PPP1R3C expression in serous ovarian cancer.

## 1. Introduction

Ovarian cancer (OC) is the seventh most common type of malignant neoplasm worldwide, and the second leading cause of gynecologic cancer death in women [1]. According to the World Health Organization (WHO), around 225,500 cases of diagnosed ovarian cancer and around 140,200 patients succumb to this disease every year [2].

OC includes a group of heterogeneous neoplasms with different morphological and biological characteristics. On these bases, three groups can be identified: epithelial ovarian carcinomas (90%), germ cell tumors (5%), and stromal tumors (4%) [3]. According to cell type, WHO classifies epithelial ovarian carcinomas (EOC) into several morphological categories: serous carcinomas, mucinous carcinomas, endometrioid carcinomas, clear cell carcinomas, transitional-cell Brenner tumors, mixed, and undifferentiated type [4]. Ovarian cancer can be further divided in Type I and Type II. In Type I, we can find low-grade serous, mucinous, endometrioid, clear cell carcinomas, and these tend to grow more slowly, often from an identifiable precursor. On the other hand, Type II tumors are characterized by high-grade and rapidly progressive disease and most cancers in this group are high-grade serous carcinomas (HGSOC) which also represents the most common histotype of all the EOCs (75%) [5].

The standard therapy for ovarian cancer includes a carboplatin (CBDCA) area under the curve (AUC) 5–6 as monotherapy for 6 cycles, or the combination CBDCA AUC 5 + paclitaxel 175 mg/m^2^ for 3–6 cycles (https://www.aiom.it/wp-content/uploads/2019/10/2019_LG_AIOM_Ovaio.pdf, Italian Association of Medical Oncology ovarian guidelines, accessed on 7 July 2023). Bevacizumab is used as a second line and maintenance treatment as an angiogenesis inhibitor [6].

Despite the best upfront treatment approach, the recurrence rate of ovarian cancer over three years following the end of first-line chemotherapy remains consistently high (75–80%) (https://www.aiom.it/wp-content/uploads/2019/10/2019_LG_AIOM_Ovaio.pdf, Italian Association of Medical Oncology ovarian guidelines, accessed on 7 July 2023). The lack of effective screening and successful treatments contribute to the high mortality and poor patient prognoses with advanced ovarian cancer [7]. The mechanisms involved in chemoresistance are still unclear; however, many studies suggest cancer stem cells (CSCs) as main players [8,9]. CSCs are a tumor cells subpopulation responsible for tumor initiation, treatments failure and cancer relapse, which make them strongly related to patients’ survival [9,10].

Their role in chemoresistance is not completely clear; however, many different factors seem to participate to their drug resistance. For instance, they have high activity of telomeric components, protective autophagy machineries, and hypoxia that plays a role in promoting epithelial-to-mesenchymal transition [8]. Moreover, their transition between stem- and non-stem, and between quiescence and proliferation states, leads to a heterogeneous population of cells, which drives the tumor to easily adapt with changes in the tumor microenvironment [11]. Several signaling pathways are also implicated in drug resistance, such as TGF-β and Notch, whose co-activation causes CSCs exhibiting phenotypic plasticity [8].

Advances in high-throughput technologies allowed the development of several approaches for the discovery of novel biomarkers. Furthermore, copy number alteration (CNA) analysis has been recently linked to gene signatures that predict adverse prognoses across multiple types of cancer [12,13,14]. In fact, copy number alterations contribute to genomic heterogeneity. CNA signature analysis introduces an innovative approach to understanding the intricate nature of genomics. It provides a sophisticated framework for investigating CNA patterns at a molecular level, offering valuable insights into the genomic mechanisms underlying certain types of cancer. Moreover, this method can potentially uncover therapeutic targets and establish prognostic associations that can aid in the development of more effective treatments [15].

In ovarian cancer, the presence of extensive CNAs is reported as a characteristic of high-grade endometrioid tumors [16]. Moreover, data from more than 600 cases of HGSOC revealed that this type of OC encompasses a spectrum of genomes influenced by multiple mutational processes, resulting in distinct patterns of genomic abnormalities. Interestingly, by quantifying the exposure to specific copy number (CN) patterns at the time of diagnosis, they predicted both the overall survival (OS) of patients, and the likelihood of relapse with resistance to platinum-based treatments [17]. Another study conducted on ovarian mucinous cystadenocarcinoma and clear cell adenocarcinoma cell lines highlighted amplifications and deletions in distinct genomic regions. Additionally, they revealed the prognostic significance of some genes in ovarian cancer, particularly the positive impact of their expression on overall survival [18].

**Figure 1:**
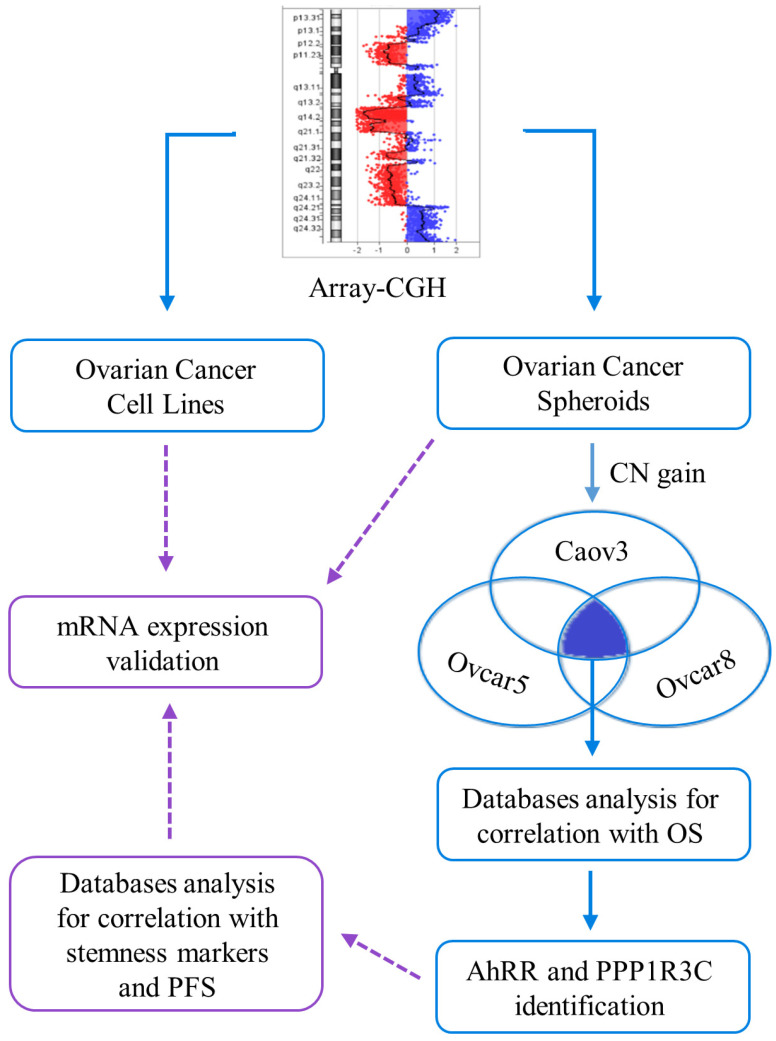
Graphical abstract. Blue: phase I of the study; violet: phase II.

In this study, we analyzed genes involved in copy number gains in our ovarian cancer stem cells (represented by ovarian cancer spheroids) and in the corresponding cell lines, with the aim to correlate their expression with patients’ follow-up in order to identify potential prognostic genes for serous ovarian cancer (Figure 1).

## 2. Results

### 2.1. Ovarian Cancer Spheroids Are Characterized by Stemness Markers’ Expression, Clonogenic Nature, and Peculiar Pathways Activation

**Figure 2:**
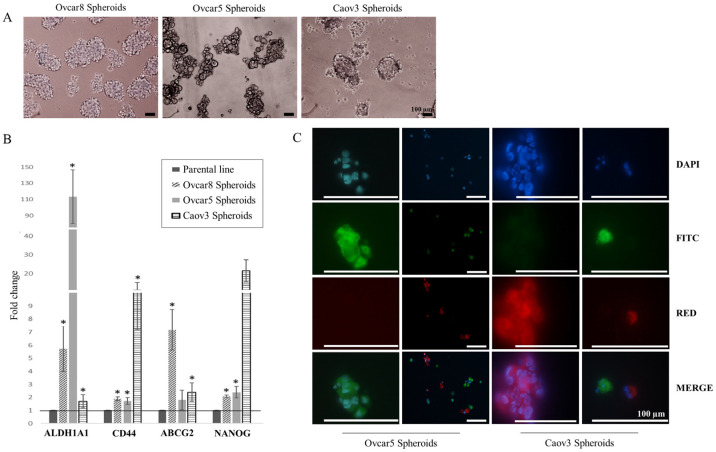
(**A**) Spheroids established from three different cell lines. Scale bar 100 µm. (**B**) Ovarian cancer stemness markers’ expression levels: each value is expressed in terms of fold change between a spheroid and its corresponding cell line. Statistical significance was determined by Student’s t-test (* *p*-value < 0.05). (**C**) Ovarian cancer spheroid PKH staining: half the spheroid population was labeled with FITC, and the other with RED, to follow label transfer during cell division. The MERGE panel shows the presence of just one of them, thus confirming cells origin from a single cell. Scale bar 100 µm.

Ovarian cancer spheroids were obtained from three ovarian cancer cell lines: Ovcar5, Ovcar8, and Caov3, representative of high-grade serous histotype, by an anchorage-independent culture system (Figure 2A, see Section 4 for details). Firstly, we analyzed the expression of known ovarian cancer stemness markers: ALDH1, CD44, ABCG2, and NANOG [8,9,19,20,21,22,23]. All ovarian cancer spheroids exhibited higher expression of these markers compared to the corresponding cell line. Particularly, Ovcar5, Caov3, and Ovcar8 spheroids showed high levels of ALDH1, CD44, and ABCG2, respectively (Figure 2B).

Additionally, to explore spheroids’ clonogenic nature, we performed PKH assay on Ovcar5 and Caov3-derived spheroids. This assay allows distinguishing spheroids from cell aggregates. Results showed that all spheroids were generated from single cells; in fact, each clone was stained with only one fluorochrome and not with both, thus confirming their clonal nature (Figure 2C).

**Figure 3:**
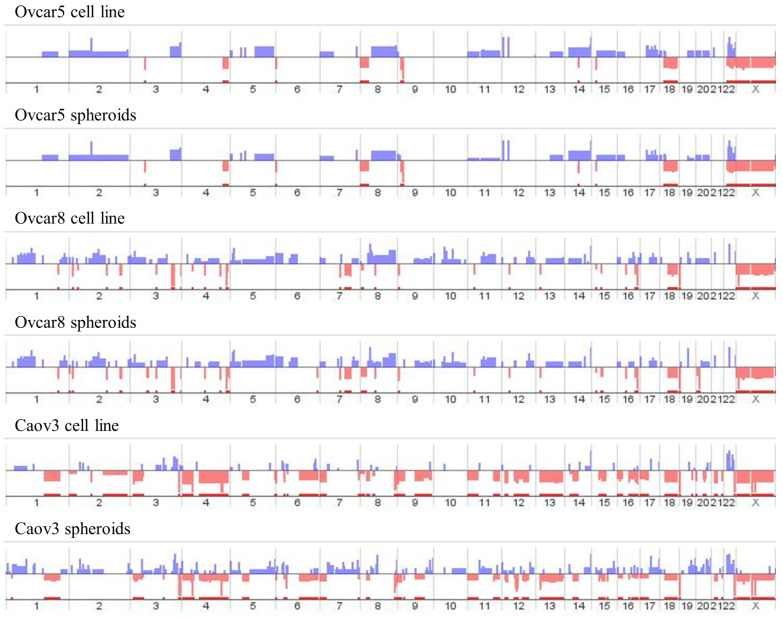
Array-CGH results. Copy number gains (blue) and losses (red) for all samples are reported. *x*-axis indicates the chromosomes; *y*-axis indicates the log2ratio values.

To characterize cell lines and their respective spheroids at genomic level, we performed a preliminary array comparative genomic hybridization (array-CGH) analysis (Figure 3).

Next, we investigated which genes were involved in copy number alterations and their respective pathways, using the DAVID Functional Annotation Bioinformatics Microarray Analysis (KEGG_pathways function). Regarding statistically significant cancer related pathways (*p* < 0.05), we underlined those shared among the cell lines and their respective spheroids and those that were exclusive, performing a preliminary study of both of these.

Particularly, in Caov3 spheres, the KEGG_pathways analysis revealed a copy number gain in genes involved in chemical carcinogenesis, specifically in EGF, FGF, and DLL (Notch homologous), that increased CSC activity, proliferation, and migration. Moreover, they showed an enhanced activity in drug metabolism, mostly linked to cytochrome P450 thus translating, as expected, to a lower drug sensitivity. Interestingly, the Caov3 cell line showed a copy number loss in genes involved in the FoxO signaling pathway, a pathway involved in the cell cycle, apoptosis, and autophagy, which was not found in the spheroids. The same was found concerning cell adhesion molecules and the AMPK signaling function, involved in the cell cycle, mTOR, and autophagy. Ovcar8 spheroids showed a copy number gain in genes involved in pathways, mainly linked to angiogenesis, proliferation and survival, migration, and invasion. In addition, the analysis showed that the basal transcription factors pathways for the RNA Polymerase II were in gain. Ovcar5 spheroids exhibited a lower functionality of some molecules involved in the platinum drug resistance pathway, specifically BID, CASP3, NOXA and CDKN2A. These alterations lead to a reduced pro-apoptotic effect and to a lower cell cycle regulation, which is coherent with CSCs characteristics.

Taken together, these preliminary results allowed us to validate our model as ovarian cancer stem cells, evidencing some CSCs characteristics that may also explain the spheres’ lower sensitivity to standard chemotherapy.

### 2.2. AHRR, GALNT10, and PPP1R3C Expression Correlates with Patients’ Overall Survival

Subsequently, we focused on genes involved in copy number gains (Figure 3, blue lines) shared by all spheroids. No CN gains shared by all spheroids were found. In most cases, the corresponding cell line showed the same alteration. In order to evaluate the potential role of the selected genes in ovarian cancer prognosis, we analyzed the correlation of their expression to patients’ survival in different web servers (The Human Protein Atlas, https://www.proteinatlas.org/ (accessed on 5 May 2023); OncoDB, https://oncodb.org/ (accessed on 5 May 2023); Gene Expression Profiling Interactive Analysis 2 (GEPIA2), http://gepia2.cancer-pku.cn/#index (accessed on 5 May 2023); and Kaplan–Meier Plotter, https://kmplot.com/analysis/ (accessed on 5 May 2023)). All these servers analyze TCGA data that are referred to serous histotype cancers. Genes with no significant correlation in all the examined databases were excluded; on the other hand, genes whose expression was negatively correlated with patients’ overall survival across all databases were selected for the subsequent studies (Table 1). In particular, AhRR, GALNT10 and PPP1R3C showed a strong correlation with prognosis (Table 1).

However, since the role of GALNT10 in ovarian cancer had already been reported [24,25], we focused our attention on AhRR and PPP1R3C.

### 2.3. AhRR and PPP3R1C Expression Correlates with Patients’ Worse Prognoses

**Figure 4:**
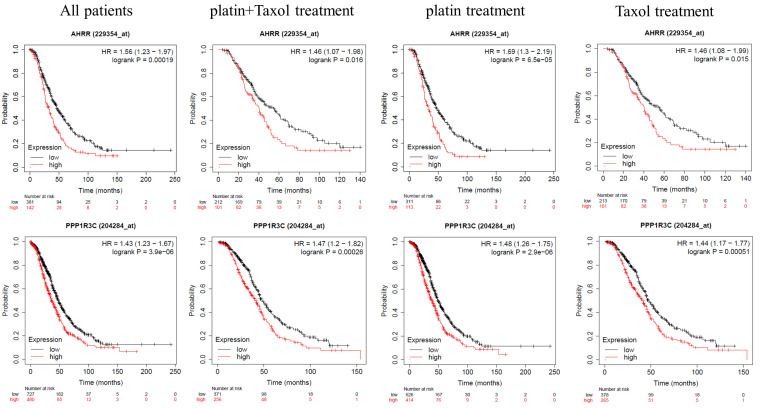
Kaplan–Meier Plotter correlation between patients’ overall survival and AhRR, PPP1R3C expression (TCGA and GSE datasets). Correlation between, respectively (left to right), all patients, patients treated with taxol + platin, platin treated, and taxol treated patients’ overall survival, and AhRR (**top panel**) and PPP1R3C (**bottom panel**) expression.

To investigate the role of AhRR and PPP1R3C in ovarian cancer, we analyzed the data from The Cancer Genome Atlas (TCGA) and GSE datasets using Kaplan–Meier Plotter web server (https://kmplot.com/analysis/index.php?p=service&cancer=ovar (accessed on 5 May 2023)), selecting the serous histotype. For both genes, correlation with patients’ overall survival remained significant, even when dividing patients into different groups, namely: treated with Taxol, platin, or a combination of these two drugs (Figure 4).

**Figure 5:**
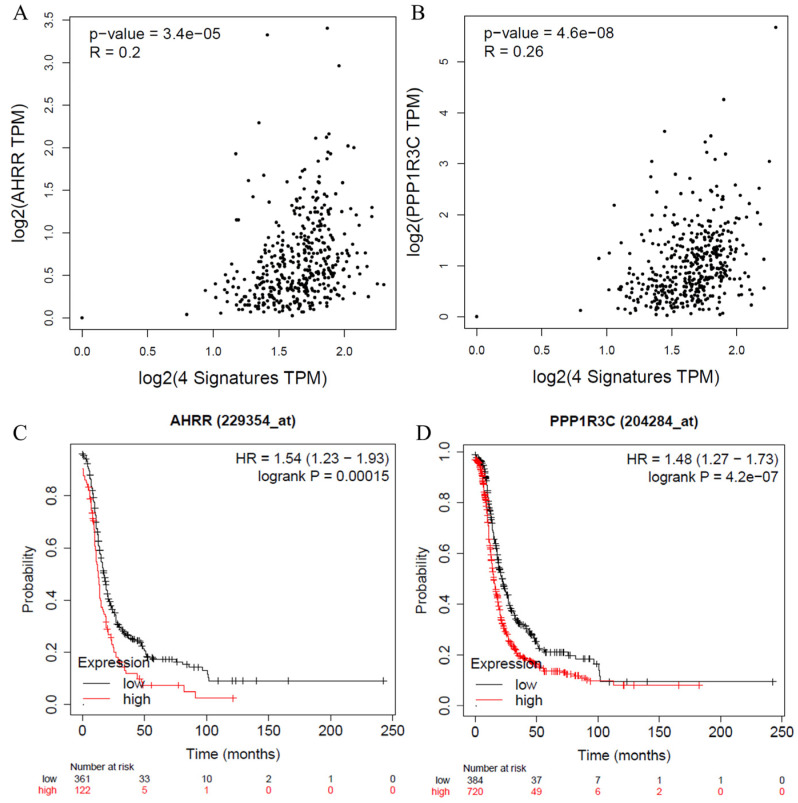
GEPIA2 correlation between AhRR (**A**) and PPP1R3C (**B**) expression and stemness markers’ (ALDH1A1, CD44, ABCG2, NANOG) expressions (4 signatures) in ovarian cancer tissue (TCGA_OV) (*p*-value < 0.01). TPM: transcripts per million. Kaplan–Meier Plotter correlation between patients’ progression-free survival and AhRR (**C**) or PPP1R3C (**D**) expression (TCGA and GSE datasets) (*p*-value < 0.01).

Based on these results, we examined TCGA dataset using GEPIA2 web server to determine whether the expression of AhRR and PPP1R3C correlates with the expression of cancer stem cells markers. Interestingly, AhRR and PPP1R3C expression significantly correlates with stemness markers’ (ALDH1A1, CD44, ABCG2, NANOG) expressions in ovarian cancer tissues (*p* < 0.01) (Figure 5A,B). Altogether, these data may suggest a potential prognostic role of AhRR and PPP1R3C, independent from chemotherapy treatment, in ovarian cancer.

To better analyze AhRR and PPP1R3C’s role in treatment responses, we investigated whether AhRR and PPP1R3C expression also correlates with ovarian cancer patients’ progression-free survival (PFS). TCGA data and GSE datasets (Kaplan–Meier Plotter web server) analysis revealed a negative significant correlation between higher expression of both AhRR and PPP1R3C, and PFS (Figure 5C,D). Interestingly, this correlation was retained independently from the treatment, as observed with OS results. Concerning surgery, AhRR expression seemed to be less important in cases with optimal surgery; in fact, it was not related to OS (*p* > 0.05, Figure S1) and correlation with PFS showed a borderline *p*-value (*p* = 0.047, Figure S1). PPP1R3C higher expression, instead, correlated with worse OS and PFS, independently from debulking surgery (Figure S1).

To also evaluate this correlation in our CSCs, we performed a real-time PCR. Our results showed a statistically significant increase in AhRR expression in Ovcar5 and Ovcar8 cell lines, and derived CSCs, but not in Caov3. PPP1R3C expression was enhanced only in the Ovcar5 cell line and its spheroids (Table 2).

**Figure 6:**
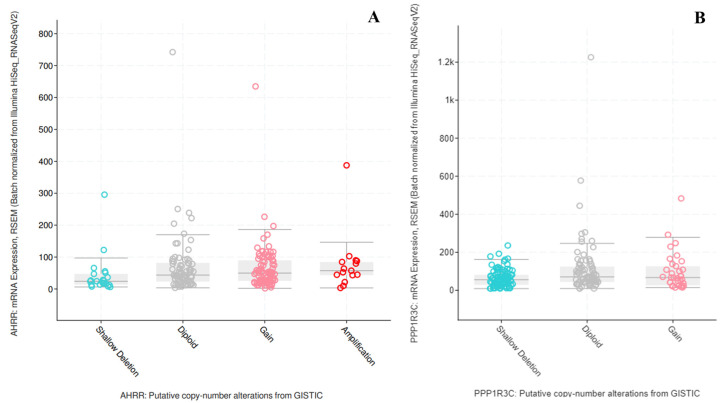
Correlation between CNAs and mRNA expression for AhRR (**A**) and PPP1R3C (**B**) on TCGA data. One-way ANOVA, *p* > 0.05.

Taken together, these results evidenced a lack of correlation between CNAs and mRNA expression for these genes, also confirmed by the TCGA data (https://www.cbioportal.org/, accessed on 5 May 2023), one-way ANOVA *p* > 0.05, Figure 6). Moreover, the presence of CNAs does not appear to be prognostic (https://portal.gdc.cancer.gov/, accessed on 5 May 2023), Chi-square test *p* > 0.05 for 6-month, 1-year, 5-year- and overall survival, Table 3). However, AhRR and PPP1R3C’s increased expression were maintained in CSCs subpopulations of two and one line, respectively.

### 2.4. AhRR and PPP1R3C Expression Correlates with Prognosis in Other Cancers

To investigate whether AhRR and PPP1R3C expression correlates with a worse outcome of patients in other cancers, we analyzed all TCGA datasets using GEPIA2 web server (accessed on 5 May 2023). While high AhRR expression significantly correlates with worse overall patient survival in different cancers (breast carcinoma, chromophobe renal cell carcinoma, lower grade glioma, sarcomas, and nevi and uveal melanomas, see Figure S2), PPP1R3C expression showed a negative or positive prognostic role, based on the cancer type (Figure S3). No TCGA dataset showed a positive prognosis linked to a high AhRR expression.

## 3. Discussion

One of the main reasons that ovarian cancer is the second leading cause of death for gynecological cancer is the lack of effective treatments linked to chemoresistance [1,3]. Recently, many studies have reported the central role of CSCs in chemoresistance and then in patients’ prognoses; however, no significant improvements have been made [5]. The use of biomarkers in diagnosis, therapy and prognosis has gained increasing interest over the last decades. Specifically, biomarker analysis in cancer patients is necessary to assess the risk of disease progression and subsequent relapse following therapy [5].

Genomic imbalances also hold great significance in cancer prognosis. In fact, rapidly progressing DNA microarray technologies allow the detection of pathogenic copy number changes in the genome, with high resolution and efficiency in identifying genes involved in cancer proliferation, progression, and metastasis [12,26,27,28].

Based on these considerations, this study aims to analyze the genes involved in copy number alterations in three cell lines and their derived CSC subpopulations, in order to identify genes potentially involved in ovarian cancer patients’ prognoses (Figure 1).

We validated our ovarian cancer spheroids through stemness markers’ expressions, clonogenic capacity (Figure 2), and preliminary array-CGH analysis. These findings underline pathways involved in CSC proliferation, metastasis, and drug resistance that are completely consistent with the characteristics of CSCs, which include a natural resistance and adaptability to external insults [29].

Subsequently, we focused our attention on CN gains shared by all spheroids, because their occurrence could suggest a role in ovarian cancer prognosis. A deeper database analysis of genes involved in copy number gains revealed three genes whose expression negatively correlates with ovarian cancer patients’ overall survival in all tested datasets: AhRR, GALNT10, and PPP1R3C (Table 1). The role of GALNT10 in ovarian CSCs, drug resistance, and patients’ prognoses has been already reported in several works [24,25], so we focused our attention on AhRR and PPP1R3C.

The aryl hydrocarbon receptor repressor is involved in the Ahr/CYP1 pathway, by competing with the cytosolic receptor AhR (aryl hydrocarbon receptor) for heterodimer formation with the aryl hydrocarbon receptor nuclear translocator (ARNT), resulting in the repression of AhR and a subsequent binding of AhRR to the xenobiotic response element (XRE). Furthermore, several studies hypothesized the Ahr/AhRR role in CSC proliferation and renewal. However, how AhRR influences cancer progression is not well defined; in fact, it seems to act according to the tumor type, either as activator or as suppressor [30].

AhRR hypermethylation and its subsequent silencing is reported to be involved in enhanced growth potential in lung cancer cells; moreover, its repression may also lead to an aggressive tumorigenic phenotype. AhRR hypermethylation seems to also occur in other cancer conditions, including ovarian cancer [31]; however, our databases analyses reported different AhRR expression levels in ovarian cancer samples, and a correlation between higher AhRR expression levels and patients’ prognoses (Figure 4).

Coherently with this latter evidence, in head and neck cancer, a higher expression of AhRR correlates with a higher production of VEGFD, upregulation of Akt, and subsequent tumor growth [32]. Moreover, AhRR overexpression in colon cancer leads to cell proliferation and altered cell adhesion, thus enhancing metastatic properties [33].

Protein phosphatase 1 regulatory subunit 3C (PPP1R3C) is a regulator of PP1, and it activates glycogen synthase, reduces glycogen phosphorylase activity, and limits glycogen breakdown. In cancer, PPP1R3C is reported to act as a tumor suppressor, and to be highly methylated in cervical cancer [34] and melanoma [35], thus resulting in cancer cell proliferation associated with high glucose levels in blood. Concerning melanoma, this data is also confirmed in TCGA, where a lower expression of PPP1R3C is related to a worse overall survival (Figure S3). On the other hand, in renal cell carcinoma, PPP1R3C is overexpressed and seems to be a cancer promoter [36]. For colorectal carcinoma, two different studies reported distinct roles of PPP1R3C: on one hand, it is linked to an aggressive phenotype following its methylation [37]; on the other, coherent with data from TCGA (Figure S3), it is reported to be poorly methylated, and its consequent overexpression correlates with a higher proliferation of colon cancer cells [38]. To date, no data are reported about the potential role of PPP1R3C in ovarian cancer.

Our study demonstrates that a higher expression level of PPP1R3C was related to poor patient prognoses and, as for AhRR, the overall survival was not influenced by the therapeutic strategy (Figure 3). On top of that, the positive correlation between AhRR and PPP1R3C expression and ovarian cancer stemness markers’ expressions (Figure 5) suggest a potential role in cancer stem cells, and reinforces their use as prognostic markers. Furthermore, several studies reported that AhR repression is strongly needed for the preservation of stemness properties; in fact, it seems necessary to maintain embryonic stem cell mitotic progression and prevent premature loss of pluripotency [39].

This suggestion is further supported by a significant correlation between AhRR and PPP1R3C expression levels and patients PFS (Figure 5). Progression-free survival is defined as the time from randomization or initiation of treatment to the occurrence of disease progression or death [40], giving us information about treatments’ ability to eliminate all cancer cells, including the ones with an evolutionary advantage represented most of the time by cancer stem cells.

Furthermore, it has been previously reported that higher cancer stemness marker expressions were related to worse PFS in ovarian cancer patients [41], thus strengthening the potential for AhRR and PPP1R3C involvement in cancer stem cell regulation.

For this reason, we checked the mRNA expression of AhRR and PPP1R3C in our spheroids (Table 2). Unfortunately, no correlation between copy number gain and expression level was found. This lack of correlation was further confirmed by TCGA data analysis (Figure 6), suggesting a pivotal role of epigenetic mechanisms. Additionally, the presence of CNA is not related to worse prognosis of carriers (Table 3). Interestingly, GDC Data Portal analysis revealed a high frequency of AhRR CN gain in the TCGA-OV cohort (51% of patients). This high frequency may be the consequence of the chromosomal localization; in fact, the *AhRR* gene, located on chromosome 5, shares the same chromosomal band (5p15.33) with the telomerase reverse transcriptase (*TERT*) gene. It is well known that cancer cells become immortalized through telomere maintenance mechanisms, such as TERT activation [42], following cancer-specific genetic alterations such as copy number gain and recurrent promoter mutations [43].

Finally, AhRR and PPP1R3C’s correlation with patients’ worse OS was identified in other cancers (Figures S2 and S3). These observations can provide a proof-of-concept for additional studies and corroborate their important role in cancer.

In conclusion, we reported for the first time, to the best of our knowledge, the prognostic role of AhRR and PPP1R3C expression in serous ovarian cancer, and their correlation with ovarian cancer stem cell markers. We also demonstrated that increased AhRR and PPP1R3C expression was maintained in some CSCs subpopulations, suggesting their possible role in ovarian cancer. However, due to the limited number of analyzed samples, additional study will be necessary to better define the potential role of these genes in CSC subpopulations.

## 4. Materials and Methods

### 4.1. Cell Lines

Ovarian cancer cell lines Caov3, Ovcar5, and Ovcar8, were purchased from ATCC (American Type Culture Collection, Manassas, VA, USA) and Sigma-Aldrich (St. Louis, MO, USA). Caov3 were grown in Dulbecco’s Modified Eagle’s Medium (DMEM, EuroClone, Milano, Italy), completed with the addition of 10% fetal bovine serum (FBS, EuroClone, Milano, Italy). Ovcar5 and Ovcar8 were grown in RPMI 1640 with the addition of 10% FBS. All the culture media had 1% Penicillin-Streptomycin (EuroClone, Milano, Italy) added. All these cell lines were kept in an incubator, in a humidified atmosphere at 5% CO_2_ and 37 °C.

### 4.2. Ovarian Cancer Spheroids

Ovarian cancer spheroids were generated following an anchorage independent growth assay starting from three different cell lines: Caov3, Ovcar5 and Ovcar8. Ovarian cancer cell lines (10^6^/mL) were seeded in ultra-low attachment plates in Dulbecco’s Modified Eagle’s Medium (DMEM) F-12 (Euroclone, Milano, Italy) with 1% Penicillin/Streptomycin (Euroclone, Milano, Italy), and completed with B27 supplement (2.5 mL/L, Life Technologies, Carlsbad, CA, USA), epidermal growth factor (EGF, 20 ng/mL, Miltenyi Biotec, Singapore), and basic fibroblast growth factor (bFGF, 10 ng/mL, Miltenyi Biotec, Singapore), in order to allow spheroids formation. Subsequently, spheroids were dissociated at least 5 times [44] before being characterized and used for subsequent experiments. Spheroids were maintained in a humidified atmosphere at 5% CO_2_ and 37 °C.

### 4.3. PKH Assay

All samples and respective controls were stained according to the manufacturer’s instructions (Sigma-Aldrich, St. Louis, MO, USA). Briefly, ovarian cancer spheroids were dissociated and reduced to single cells, and stained with two different PKH dyes. Half the population was resuspended in diluent C and stained with PKH2 Green Fluorescent Cell Linker, and the other half was resuspended in diluent A and stained with PKH26 Red Fluorescent Cell Linker (Sigma-Aldrich, St. Louis, MO, USA). The populations were then re-seeded together to allow spheroid formation. The sample was seeded in a chamber slide, and centrifuged at 2000 rpm for 20 min to allow cells to adhere to the slide. Finally, nuclei were counterstained with DAPI (Sigma-Aldrich, St. Louis, MO, USA) and each sample was observed with a fluorescent microscope (Nikon Eclipse Ni, Nikon, Amstelveen, The Netherlands) and captured with NIS-Elements software (v. 4.50.00, Nikon, Amstelveen, The Netherlands).

### 4.4. RNA Extraction and Real Time-PCR

Lymphocytes were extracted from the blood of four healthy donors and used as the negative control. Informed consent was obtained from all donors. Lymphocytes layers were isolated using Lympholyte Cell Separation Media (Cedarlane, Burlington, ON, Canada). Total RNA from all ovarian cancer spheroids, the corresponding cell lines and lymphocytes were isolated by RNeasy Mini Kit according to manufacturer’s instructions (QIAGEN, Hilden, Germany). RNA quantity and quality were determined with a Nanodrop ND-2000 spectrophotometer (Thermo Fisher Scientific, Waltham, MA, USA). After measuring the concentrations, total RNA was reverse-transcribed using the High Capacity cDNA Reverse Transcription Kit (Thermo Fisher Scientific, Waltham, MA, USA). For each sample, from 250 ng to 1 µg of RNA were reverse transcribed. qRT-PCR was performed using TaqMan Gene Expression Master Mix (Applied Biosystems, Waltham, MA, USA), and the plates were analyzed by thermocycler StepOnePlus Real-time PCR System (Applied Biosystems, Waltham, MA, USA). ALDH1 (Hs00946916_m1), ABCG2 (Hs01053709_m1), CD44 (Hs01075861_m1) and NANOG (Hs04260366_g1) were selected as stemness markers. AhRR (Hs01005075_m1) and PPP1R3C (Hs01921501_s1) were checked to confirm CNA’s data. All TaqMan probes were purchased from Life Technologies (Waltham, MA, USA). The relative mRNA expression was calculated by the 2-ΔΔCt method and normalized to GAPDH (Hs99999905_m1) expression.

### 4.5. Array-Comparative Genomic Hybridization (Array-CGH)

DNA was extracted from ovarian cancer spheroids and the corresponding cell lines according to the manufacturer’s instructions (QIAGEN, Hilden, Germany), using QIAamp DNA Mini Kit. The DNA was quantified using the Nanodrop ND-2000 spectrophotometer (Thermo Fisher Scientific, Waltham, MA, USA). Samples with a concentration over 10 µg/mL and an absorbance ratio A260/280 over 1.8 and A260/230 over 1.7, as required from kit’s instructions, were used for array-comparative genomic hybridization analysis. A total of 500 ng of each sample were used for the analysis.

Array-CGH analysis was performed using SurePrint G3 Human CGH Microarray 8 × 60 K (Agilent Technologies, Santa Clara, CA, USA) according to the manufacturer’s instructions. The arrays were scanned at 2-μm resolution and analyzed using Agilent Feature Extraction and Agilent Cytogenomics v5.2 software (Agilent Technologies, Santa Clara, CA, USA).

The estimated percentage of mosaicism was calculated using the formula determined by Cheung SW et al. [45]. In particular, non-mosaic gains and losses were identified by standard log2 ratio values for all samples: values over 0.6, which correspond to three copies, identify non-mosaic gains; values under −1, which correspond to 1 copy, identify non-mosaic losses. Accordingly, log2 ratio values for mosaic gains range between the DLRS (derivative log ratio spread) value and 0.6 and for mosaic losses between the DLRS value and −1. Amplifications and deletions were identified by values over +1 and under −1.7, respectively [46].

### 4.6. Bioinformatic Analyses

#### 4.6.1. Analysis of Genes Involved in Copy Number Alterations and Their Respective Pathways

DAVID Functional Annotation Bioinformatics Microarray Analysis (https://david.ncifcrf.gov/home.jsp, accessed on: 13 September 2022) was used for the analysis of the genes involved in CNAs and the respective altered pathways. The pathways were analyzed using the function “KEGG_PATHWAYS database”. The results of the cell lines were then compared with the respective spheres. For the purpose of the study, we only considered cancer-related pathways (*p*-value < 0.05).

#### 4.6.2. Analysis of AhRR, GALNT10 and PPP1R3C Expression in Ovarian Cancer

The Human Protein Atlas (https://www.proteinatlas.org/, accessed on: 5 May 2023), OncoDB (https://oncodb.org/, accessed on: 5 May 2023), Kaplan–Meier plotter (https://kmplot.com/analysis/, accessed on: 5 May 2023), and GEPIA2 (http://gepia2.cancer-pku.cn/, accessed on: 5 May 2023) web servers were used to analyze the correlation between the selected genes’ expressions and patients’ overall survival in ovarian cancer (Table 1). Based on the mRNA values, patients were classified into two groups: “low” (under cut off) or “high” (over cut off) expression. Selected cut-off value was “best expression cut-off” (The Human Protein Atlas), “50% cut-off” (OncoDB), “auto selected best cut-off” (Kaplan–Meier plotter), and “median cut-off” (GEPIA2). *p*-value < 0.05 was considered statistically significant.

The Cancer Genome Atlas and the GSE dataset (GSE14764, GSE15622, GSE18520, GSE19829, GSE23554, GSE26193, GSE26712, GSE27651, GSE30161, GSE3149, GSE51373 GSE63885, GSE65986, GSE9891) were used to estimate ovarian cancer patients’ overall survival and progression-free survival, based on Gene Chip mRNA expression data. To elaborate the data from the databases, the Kaplan–Meier plotter was used. Based on the sample that was being considered (all serous patients/platin treated/Taxol treated/Taxol + platin treated patients) the parameter “Restrict analysis to treatment groups—Chemotherapy”, was set. *p*-value < 0.05 was considered statistically significant.

#### 4.6.3. Correlation Analysis of AhRR and PPP1R3C with Stemness Markers

The GEPIA2 web server was used to evaluate the correlation between AhRR and PPP1R3C, and CD44, ALDH1A1, NANOG, and ABCG2 (4 Signatures) expression. Data from TCGA were used for the correlation in cancer tissues using the ‘correlation analysis’ function. Data were examined using two different correlation coefficients: Pearson and Spearman. *p*-value < 0.05 was considered statistically significant.

#### 4.6.4. Correlation between CNAs and mRNA Expression for AHRR and PPP1R3C

The cBioPortal for Cancer Genomics (https://www.cbioportal.org/, accessed on: 5 May 2023) was used to evaluate the correlation between CNAs and mRNA expression for AhRR and PPP1R3C in ovarian cancer using the plots window. One-way analysis of variance (ANOVA) had a *p* > 0.05.

CNAs’ data from human ovarian cancer samples were obtained from GDC Data Portal (https://portal.gdc.cancer.gov/, accessed on: 5 May 2023). Number of alive and dead patients was obtained by the Vital Status field (TCGA-OV), and the survival of CNA carriers was compared to all patients’ survival (Chi-square test). Time-point survival was achieved by setting different “Days to the Dead” field.

#### 4.6.5. Analysis of AHRR and PPP1R3C Expression in Other Cancers

The GEPIA2 web server was used to analyze the correlation between selected genes’ expression in different cancers with patients’ OS (TCGA datasets). *p*-value < 0.05 was considered statistically significant.

## Figures and Tables

**Table 1:** Correlation of mRNA expression and patients’ overall survival.

	The Human Protein Atlas	OncoDB	GEPIA2	Kaplan–Meier Plotter (RNA-Seq Data)	Kaplan–Meier Plotter (Gene CHIP Data)
Copy number gains shared among all spheroids and all cell lines					
*ADRA1B*	*p* < 0.01 favorable	ns	ns	*p* < 0.01 favorable	*p* < 0.01 favorable
*AHRR*	***p*** ** < 0.01 unfavorable**	***p*** ** < 0.01 unfavorable**	***p*** ** < 0.01 unfavorable**	***p*** ** < 0.01 unfavorable**	***p*** ** < 0.01 unfavorable**
*ATP10B*	*p* < 0.05 favorable	ns	ns	*p* < 0.05 favorable	ns
*CREBRF*	ns	ns	ns	ns	***p*** ** < 0.01 unfavorable**
*EBF1*	ns	ns	ns	ns	***p*** ** < 0.01 unfavorable**
*GFPT2*	***p*** ** < 0.05 unfavorable**	***p*** ** < 0.05 unfavorable**	ns	***p*** ** < 0.05 unfavorable**	***p*** ** < 0.01 unfavorable**
*GPRIN1*	ns	ns	ns	ns	***p*** ** < 0.01 unfavorable**
*KCNMB1*	*p* < 0.05 favorable	ns	ns	ns	ns
*LSM11*	***p*** ** < 0.05 unfavorable**	ns	ns	***p*** ** < 0.05 unfavorable**	*p* < 0.05 favorable
*PROP1*	ns	ns	ns	***p*** ** < 0.05 unfavorable**	*p* < 0.05 favorable
*RASGEF1C*	ns	ns	ns	ns	*p* < 0.05 favorable
*TBC1D9B*	ns	ns	ns	ns	*p* < 0.01 favorable
Copy number gains shared among all spheroids and two cell lines					
*ADAM19*	ns	ns	ns	ns	*p* < 0.01 favorable
*ADAMTS2*	***p*** ** < 0.05 unfavorable**	ns	ns	***p*** ** < 0.05 unfavorable**	***p*** ** < 0.01 unfavorable**
*COL23A1*	***p*** ** < 0.05 unfavorable**	ns	ns	ns	***p*** ** < 0.01 unfavorable**
*CPLX2*	ns	ns	ns	ns	***p*** ** < 0.05 unfavorable**
*FABP6*	*p* < 0.05 favorable	ns	ns	*p* < 0.05 favorable	ns
*GABRA1*	na	na	na	***p*** ** < 0.01 unfavorable**	ns
*GABRA6*	na	na	na	***p*** ** < 0.01 unfavorable**	***p*** ** < 0.05 unfavorable**
*GABRB2*	ns	ns	ns	ns	***p*** ** < 0.01 unfavorable**
*GABRP*	*p* < 0.01 favorable	ns	ns	*p* < 0.01 favorable	*p* < 0.01 favorable
*GALNT10*	***p*** ** < 0.01 unfavorable**	***p*** ** < 0.05 unfavorable**	***p*** ** < 0.01 unfavorable**	***p*** ** < 0.01 unfavorable**	***p*** ** < 0.05 unfavorable**
*GLRA1*	na	ns	na	***p*** ** < 0.01 unfavorable**	*p* < 0.05 favorable
*GRIA1*	***p*** ** < 0.05 unfavorable**	ns	***p*** ** < 0.05 unfavorable**	***p*** ** < 0.05 unfavorable**	ns
*HNRNPAB*	*p* < 0.01 favorable	ns	ns	*p* < 0.01 favorable	ns
*KCNIP1*	*p* < 0.01 favorable	ns	ns	*p* < 0.01 favorable	ns
*LARP1*	ns	ns	ns	ns	*p* < 0.05 favorable
*MAMDC2*	***p*** ** < 0.05 unfavorable**	ns	ns	***p*** ** < 0.05 unfavorable**	***p*** ** < 0.05 unfavorable**
*MFAP3*	***p*** ** < 0.05 unfavorable**	ns	***p*** ** < 0.05 unfavorable**	***p*** ** < 0.05 unfavorable**	ns
*MKRN2*	ns	ns	ns	ns	***p*** ** < 0.01 unfavorable**
*MIR762HG*	na	*p* < 0.05 favorable	*p* < 0.01 favorable	na	na
*NDST1*	***p*** ** < 0.01 unfavorable**	***p*** ** < 0.01 unfavorable**	***p*** ** < 0.01 unfavorable**	***p*** ** < 0.01 unfavorable**	ns
*NEURL1*	***p*** ** < 0.05 unfavorable**	ns	ns	***p*** ** < 0.05 unfavorable**	ns
*NSD1*	ns	ns	ns	ns	***p*** ** < 0.01 unfavorable**
*PC*	***p*** ** < 0.01 unfavorable**	***p*** ** < 0.01 unfavorable**	***p*** ** < 0.01 unfavorable**	***p*** ** < 0.01 unfavorable**	ns
*RANBP17*	ns	ns	ns	ns	*p* < 0.05 favorable
*RNF130*	ns	ns	***p*** ** < 0.05 unfavorable**	ns	***p*** ** < 0.05 unfavorable**
*SFXN1*	*p* < 0.05 favorable	ns	ns	*p* < 0.05 favorable	***p*** ** < 0.05 unfavorable**
*SGCD*	ns	ns	ns	ns	ns
*SH3PXD2B*	***p*** ** < 0.05 unfavorable**	ns	ns	***p*** ** < 0.05 unfavorable**	***p*** ** < 0.05 unfavorable**
*SLIT3*	***p*** ** < 0.01 unfavorable**	ns	ns	***p*** ** < 0.05 unfavorable**	ns
*TENM2*	*p* < 0.05 favorable	ns	ns	*p* < 0.05 favorable	ns
*UBTD2*	ns	ns	ns	ns	***p*** ** < 0.01 unfavorable**
*UIMC1*	ns	ns	ns	ns	ns
*ZNF346*	ns	ns	ns	ns	***p*** ** < 0.05 unfavorable**
Copy number gains shared among all spheroids and one cell line					
*NLRP12*	***p*** ** < 0.01 unfavorable**	***p*** ** < 0.01 unfavorable**	***p*** ** < 0.01 unfavorable**	***p*** ** < 0.01 unfavorable**	ns
*PPP1R3C*	***p*** ** < 0.01 unfavorable**	***p*** ** < 0.01 unfavorable**	***p*** ** < 0.01 unfavorable**	***p*** ** < 0.01 unfavorable**	***p*** ** < 0.01 unfavorable**
*PYROXD2*	ns	ns	ns	ns	*p* < 0.01 favorable

Favorable: high expression correlates with better prognosis; unfavorable: high expression correlates with worse prognosis; ns: not statistically significant; na: not available. Bold: significant unfavorable.

**Table 2:** AhRR and PPP1R3C copy number alterations and mRNA expression in tested samples.

		Ovcar5 Line	Ovcar5 Spheroids	Ovcar8 Line	Ovcar8 Spheroids	Caov3 Line	Caov3 Spheroids
AhRR	CNA	non mosaic gain	non mosaic gain	non mosaic gain	non mosaic gain	non mosaic gain	amplification
	mRNA	* fc = 53 ± 19	* fc = 26 ± 4	* fc = 27 ± 11	* fc = 44 ± 27	fc = 0.5 ± 0.03	fc = 0.4 ± 0.1
PPP1R3C	CNA	disomy	mosaic gain (43%)	non mosaic gain	non mosaic gain	disomy	non mosaic gain
	mRNA	* fc = 28 ± 4	* fc = 24 ± 7	fc = 0.4 ± 0.2	fc = 1 ± 0.8	fc = 1.3 ± 0.15	fc = 1 ± 0.25

fc: fold change (mean ± sem) relative to controls’ mean (lymphocytes = 1). * *p* < 0.05, Student’s *t*-test.

**Table 3:** Survival data of TCGA-OV patients.

Gene	Cases with CNAs n°		6 Months Survival				1 Year Survival				5 Years Survival				Overall Survival			
			Gain		Loss		Gain		Loss		Gain		Loss		Gain		Loss	
	Gain	Loss	Alive	Dead	Alive	Dead	Alive	Dead	Alive	Dead	Alive	Dead	Alive	Dead	Alive	Dead	Alive	Dead
*AhRR*	301	43	285/301 94.7%	16/301 5.3%	42/43 97.7%	1/43 2.3%	275/301 91.4%	26/301 8.6%	37/43 86%	6/43 14%	154/301 51.2%	147/301 48.8%	22/43 51.1%	21/43 48.8%	123/301 40.8%	178/301 59.1%	20/43 46.5%	23/43 53.5%
*PPP1R3C*	94	167	90/94 95.7%	4/94 4.3%	162/167 97%	5/167 3%	86/94 91.5%	8/94 8.5%	158/167 94.6%	9/167 5.4%	45/94 47.9%	49/94 52.1%	95/167 56.9%	72/167 43.1%	38/94 40.4%	56/94 59.6%	76/167 45.5%	91/167 54.5%
GDC all cases (585)			6 months survival: 555 alive (94.9%), 30 dead (5.1%)				1 year survival: 531 alive (90.8%), 54 dead (9.2%)				5 years survival: 293 alive (50.1%), 292 dead (49.9%)				Overall survival: 236 alive (40.3%), 349 dead (59.7%)			

Not statistically significant difference between CN carriers and all cases (Chi-square test, *p* > 0.05).

## Data Availability

The data that support the findings of this study are available from the corresponding author, [D.C.], upon reasonable request.

## Supplementary Materials

The following supporting information can be downloaded at: https://www.mdpi.com/article/10.3390/ijms241411455/s1.
