# Supplementary material for: AhRR and PPP1R3C: Potential Prognostic Biomarkers for Serous Ovarian Cancer
Source: Int J Mol Sci. 2023 Jul 14;24(14):11455. doi: 10.3390/ijms241411455 (PMC10380391; doi:10.3390/ijms241411455)
Supplement: Supplementary file 1 [file ijms-24-11455-s001.zip › ijms-2470443-supplementary.pdf]

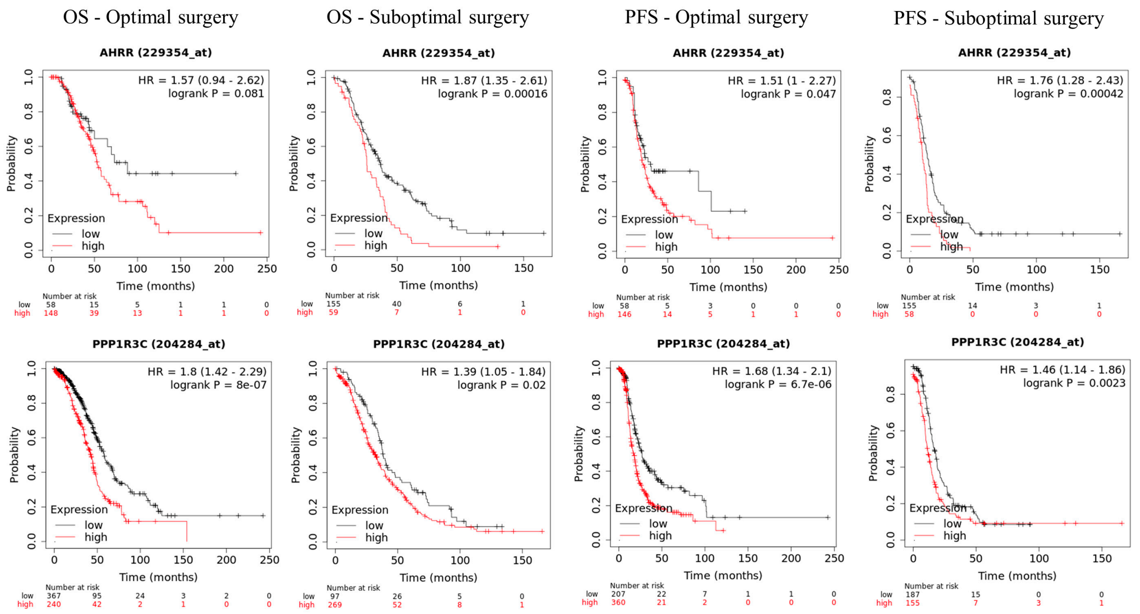

Figure S1. Kaplan–Meier Plotter correlation between patients' OS/PFS, debulking surgery and AhRR/PPP1R3C expression.

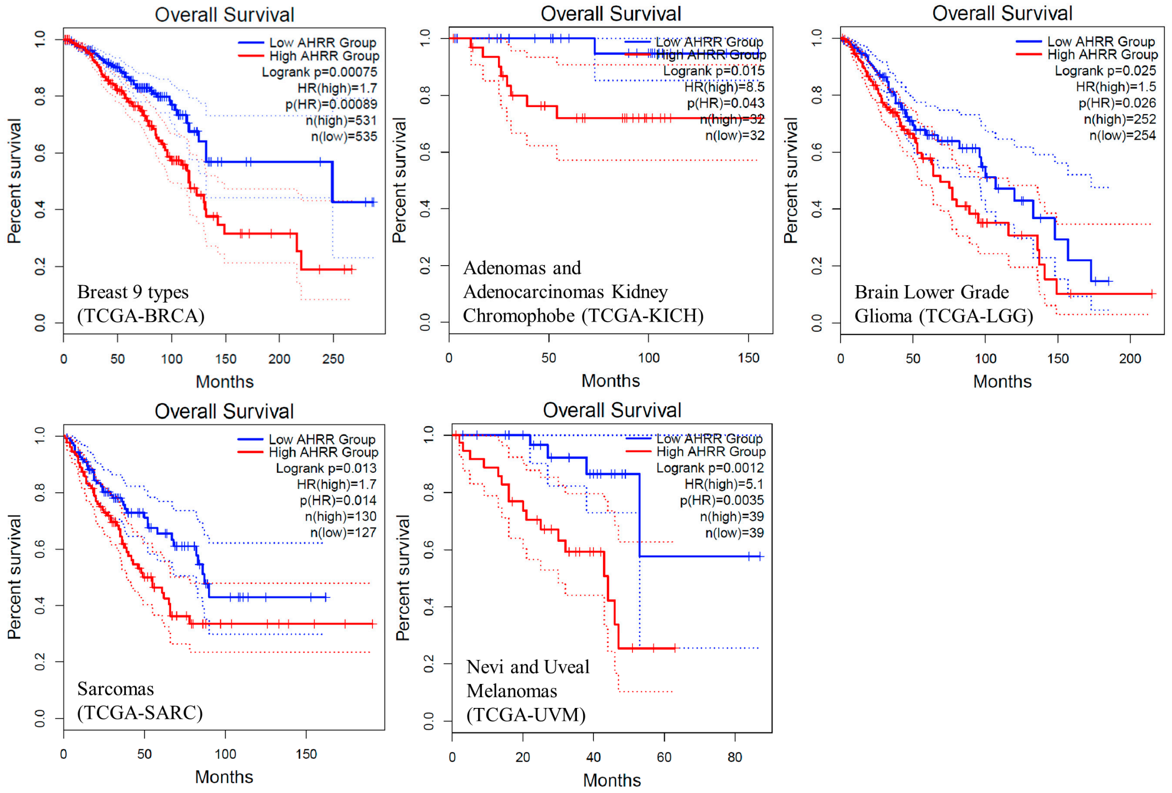

Figure S2. Relationship between patients' overall survival and AhRR expression in different TCGA datasets (GEPIA2).

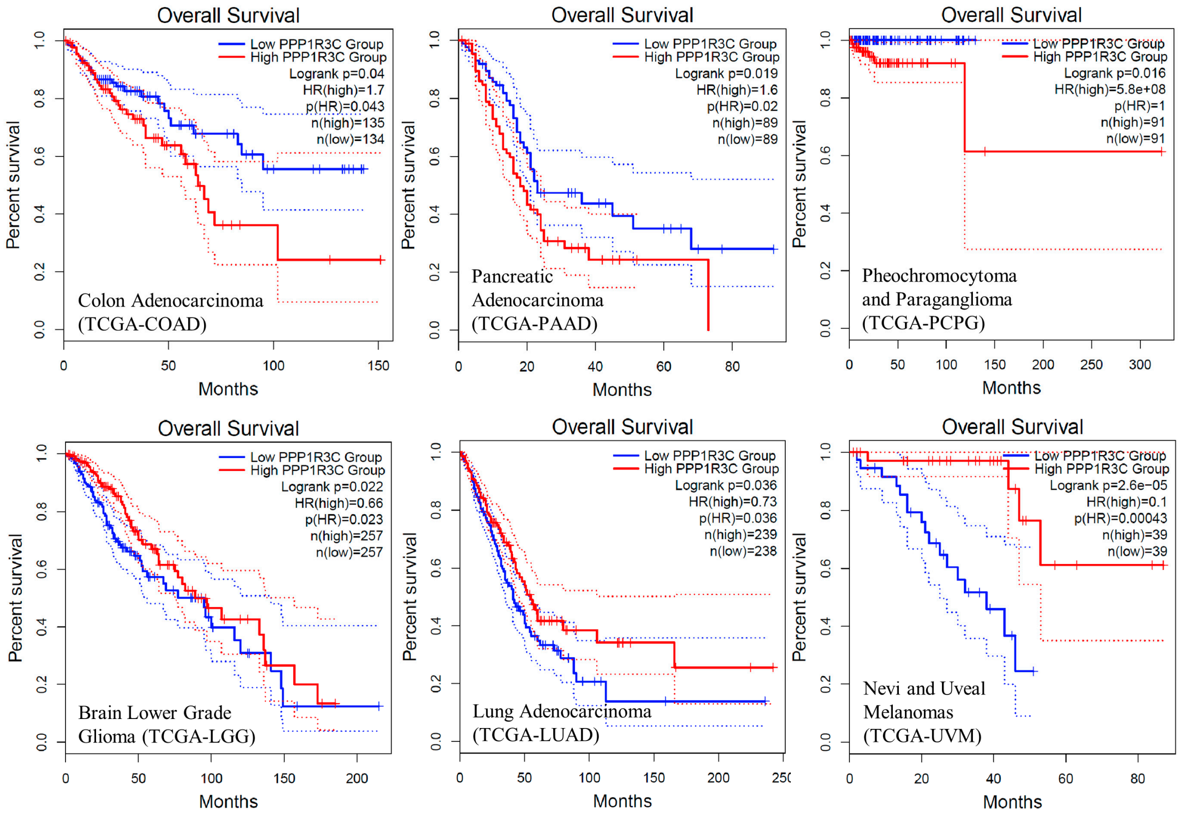

Figure S3. Relationship between patients' overall survival and PPP1R3C expression in different TCGA datasets (GEPIA2).
